# The DNMT3A R882H mutation does not cause dominant negative effects in purified mixed DNMT3A/R882H complexes

**DOI:** 10.1038/s41598-018-31635-8

**Published:** 2018-09-05

**Authors:** Max Emperle, Michael Dukatz, Stefan Kunert, Katharina Holzer, Arumugam Rajavelu, Renata Z. Jurkowska, Albert Jeltsch

**Affiliations:** 1 Department of Biochemistry, Institute of Biochemistry and Technical Biochemistry, Stuttgart University, Allmandring 31, 70569 Stuttgart, Germany; 2 Present Address: Rajiv Gandhi Center for Biotechnology (RGCB), Trivandrum, 695014 Kerala India; 3 Present Address: BioMed X Innovation Center, Im Neuenheimer Feld 583, D-69120 Heidelberg, Germany

## Abstract

The DNA methyltransferase DNMT3A R882H mutation is observed in 25% of all AML patients. DNMT3A is active as tetramer and the R882H mutation is located in one of the subunit/subunit interfaces. Previous work has reported that formation of mixed wildtype/R882H complexes leads to a strong loss of catalytic activity observed in *in vitro* DNA methylation assays (Russler-Germain *et al*., 2014, *Cancer Cell* 25:442–454). To investigate this effect further, we have prepared mixed wildtype/R882H DNMT3A complexes by incubation of individually purified subunits of the DNMT3A catalytic domain and full-length DNMT3A2. In addition, we have used a double affinity tag approach and specifically purified mixed catalytic domain complexes formed after co-expression of R882H and wildtype subunits in *E. coli* cells. Afterwards, we determined the catalytic activity of the mixed complexes and compared it to that of purified complexes only consisting of one subunit type. In both settings, the expected catalytic activities of mixed R882H/wildtype complexes were observed demonstrating an absence of a dominant negative effect of the R882H mutation in purified DNMT3A enzymes. This result suggests that heterocomplex formation of DNMT3A and R882H is unlikely to cause dominant negative effects in human cells as well. The limitations of this conclusion and its implications are discussed.

## Introduction

Epigenetic regulation conveys heritable alterations in gene expression that are not caused by changes in the primary DNA sequence^1^. There are several types of epigenetic mechanisms, including methylation and hydroxymethylation of cytosine residues in DNA (mostly in a CpG context), post-translational modifications of histone proteins and non-coding RNAs. DNA methylation is directly involved in many cellular processes by regulating genome stability, repression of repeat sequences, imprinting and cellular differentiation^2,3^. DNA methylation is introduced by enzymes called DNA methyltransferases (DNMT1, DNMT3A and DNMT3B)^4,5^ and removed with the help of TET methylcytosine dioxygenases^6^. Collectively, DNA methylation and demethylation represent critical mechanisms for cellular transformation and maintenance of cancerous cell programs^7–9^. In recent years, the rapid development of novel DNA sequencing techniques has revealed several genes that frequently contain somatic mutations in tumor tissues including DNA methylation regulators such as DNMTs and TETs^9,10^. Mutations in the DNMT3A DNA methyltransferase were reported for the first time in 2010, when it was found that a high frequency of missense mutations in DNMT3A occurs in AML^11,12^. This finding was later validated and further developed by several other groups reporting that about 25% of AML patients contain DNMT3A mutations (reviews^13,14^). Later, recurrent DNMT3A mutations have been identified in other types of cancers, including myelodysplastic syndrome (MDS) and T-cell lymphomas (reviews^13,14^). DNMT3A mutations in AML usually occur heterozygously and they show a strong enrichment of missense mutations, in particular at arginine 882 in the catalytic C-terminal domain of DNMT3A, which is most often altered to histidine.

**Figure 1:**
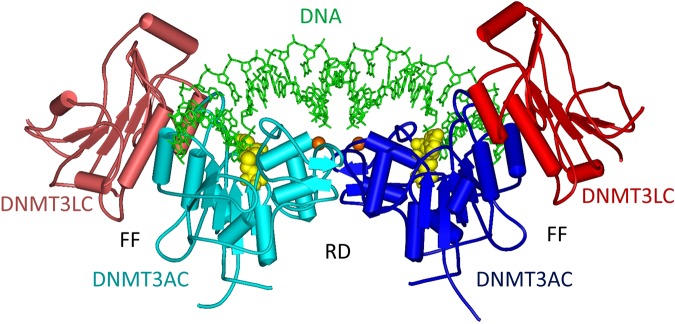
Position of the R882H mutation in the structure of the DNMT3A/DNMT3L C-terminal domain heterotetramer with bound DNA (green)^16^. The DNMT3L C-terminal domains are colored dark and light red, the DNMT3A C-terminal domains are dark and light blue. AdoMet is shown in yellow and the positions of the Cα atoms of R882 in both DNMT3A subunits are indicated by orange balls. The regions of the DNMTR3A/3A RD interface and DNMT3A/3L FF interface are annotated in black.

The structure of the C-terminal domain of DNMT3A (DNMT3AC) in complex with the C-terminal domain of its stimulator DNMT3L forms a linear heterotetramer consisting of two DNMT3L subunits (at the edges of the tetramer) and two DNMT3A molecules (in the center)^15,16^ (Fig. 1). The DNMT3A/3 L interface is called FF interface but it is also used for DNMT3A/3 A interaction in the absence of DNMT3L^17,18^. This hydrophobic interface is stable, allowing the co-purification of DNMT3A/3 L complexes^15^. The inner DNMT3A/3 A interface is called RD interface. It is a polar interface that mediates a reversible oligomerization of DNMT3A dimers (formed via the FF-interface) into a mixture of dimers, tetramers and higher aggregates depending on the buffer conditions and enzyme concentrations^17–19^. The RD interface also forms the DNA binding site of the DNMT3A complex^15,17^ indicating that a tetramer is the smallest complex, which can be catalytically active.

The R882H mutation is located in the center of the RD interface suggesting potential effects on multimerization of DNMT3A and/or DNA interaction (Fig. 1). Different studies have determined the catalytic activity of the purified R882H mutant and observed 30–70% residual activity of the mutant enzyme (Table 1)^12,20–23^. Later, studies reported that the R882H mutation exhibits a dominant negative effect in cells and *in vitro*. Kim *et al*. (2013) showed that the exogenously expressed murine R878H mutant (corresponding to human R882H) interacts with the wildtype enzyme, but it was less efficiently methylating major satellite repeats in mouse ES cells^24^. Russler-Germain *et al*. (2014) described that purified mixed enzyme preparations obtained after co-expression of wildtype and R882H in mammalian cells showed only 12% of residual activity in kinetic enzyme activity assays carried out *in vitro*. This observation led them to the conclusion that the R882H mutant has a dominant negative effect at the biochemical level and the formation of mixed complexes of wildtype and R882H subunits strongly reduces the activity of the mutant and wildtype DNMT3A subunits^22^.

**Table 1:** Compilation of catalytic activities of DNMT3A R882H reported in different studies.

Rel. activity of R882H	Reference
44%	Yamashita *et al*., Fig. 3^12^
68%	Yan *et al*., Fig. 2A (in presence of DNMT3L)^20^
54%	Holtz-Schietinger *et al*., Table 3^21^
29%	Russler-Germain *et al*., Fig. 6C^22^
62%	Emperle *et al*., Fig. 1A, 509mer DNA substrate^23^
65%	This study

## Results

**Figure 2:**
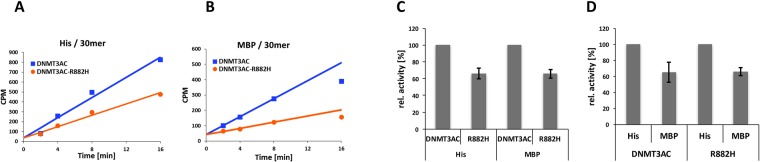
Characterization of the activity of DNMT3AC R882H homomultimers. (**A**,**B**) Exemplary data showing catalytic activities of the purified His- and MBP-tagged DNMT3AC wildtype and R882H analyzed using a 30mer DNA substrate. (**C**) Summary of the effect of the R882H mutation on the catalytic activity of His- and MBP-tagged proteins. (**D**) Summary of the effect of the MBP-tag on the catalytic activity of DNMT3AC and R882H. Error bars in (**C**,**D**) indicate the SEM based on 5 independent experiments, in which DNMT3AC and R882H were expressed, purified and assayed in parallel.

We were aiming here to investigate the mechanistic details of the dominant negative effect of the R882H mutation in mixed wildtype/R882H complexes^22^. We initially used the catalytic domain of DNMT3AC, because it can be purified with different affinity tags (see below) in good yields and sufficient quantities for the planned biochemical work^15,17^. DNMT3AC is enzymatically active^25^ and reproduces the stimulation by DNMT3L seen with full length enzymes^26^ indicating that it is a suitable model system to study multimerization and activity of DNMT3A. To start our work, we have prepared the R882H mutant in the context of His-tagged DNMT3AC and purified the proteins after recombinant expression in *E. coli*. *In vitro* methylation experiments using identical concentrations of DNMT3AC wildtype and R882H showed that the R882H mutant has a residual activity of approximately 65% with a 30 mer DNA substrate (Fig. 2A,C) that has been used as standard substrate in many of our previous publications^17,18,25,27,28^. This reduction in activity of R882H is comparable to results reported by most other groups (Table 1) in particular when considering that the relative activity of R882H is strongly dependent on the CpG site sequence context of the methylation substrate^23^.

### Formation of wildtype/R882H complexes by mixing of purified subunits does not reduce the catalytic activity

**Figure 3:**
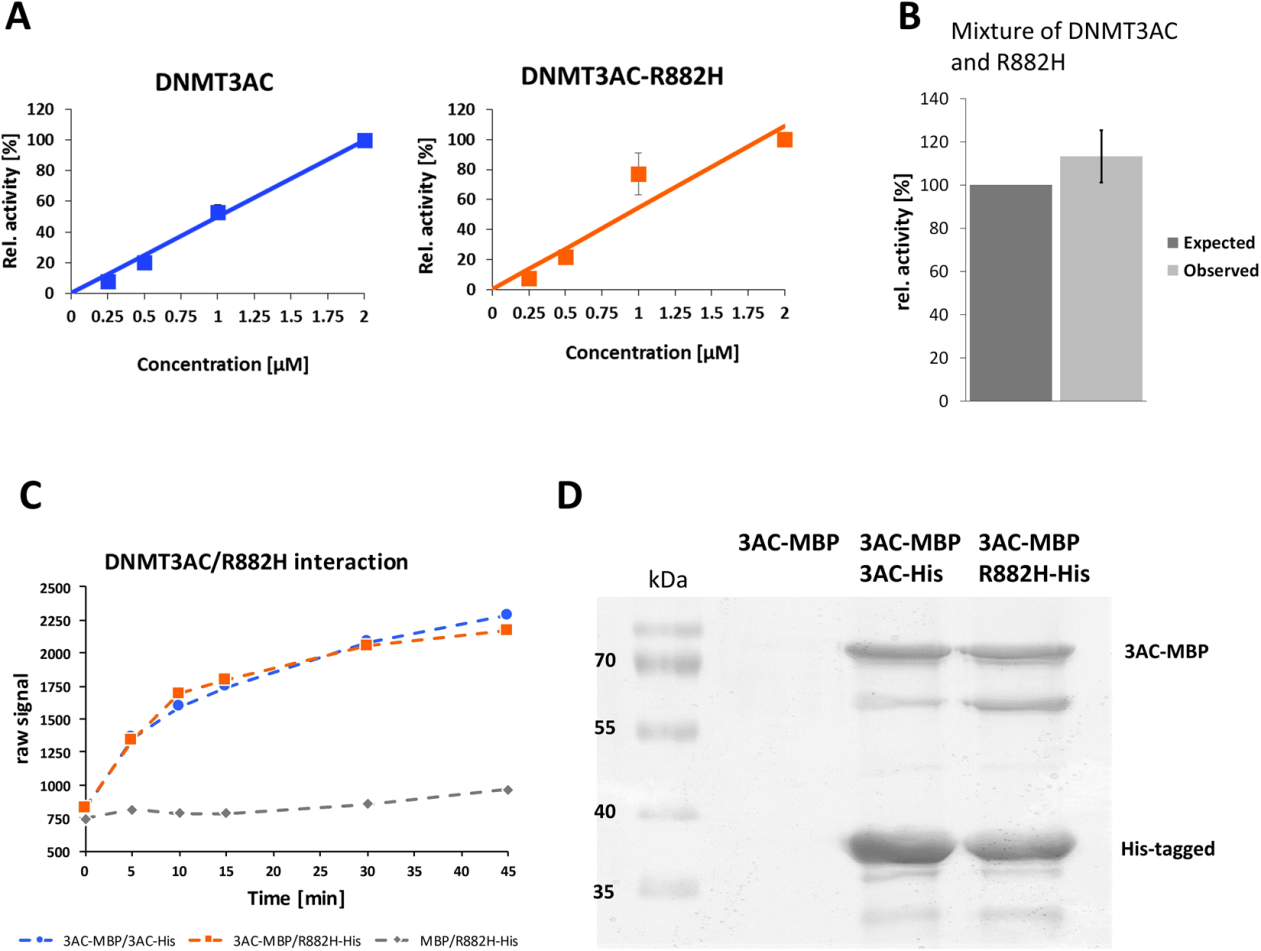
Activity of R882H/wildtype DNMT3AC complexes obtained by mixing of independently purified subunits. (**A**) The catalytic activity of wildtype and R882H DNMT3AC is linearly dependent on the enzyme concentration under the assay conditions used here. (**B**) Activity of mixed DNMT3AC/R882H preparations after pre-incubation. The dark grey bar indicates the expected activity corresponding to the sum of the activities of the two subunit types, the light grey bar shows the observed activity. The error bar indicates the SEM based on 2 independent experiments. (**C**) Documentation of heterocomplex formation after pre-incubation of MBP- and His-tagged DNMT3AC subunits. In this assay, two subunit types were used with different tags (here His- and MBP-tag). Alpha acceptor and donor beads were bound to the tags and the samples were mixed. Formation of mixed complexes leads to an approximation of the two beads causing the emission of a light signal. The image shows Alpha-screen raw data observed after mixing of His and MBP-tagged DNMT3AC (blue curve) or MBP-DNMT3AC with His-R882H (orange curve). The signal observed after mixing of MBP with His-DNMT3AC (grey curve) illustrates the background. The experiment was carried out in triplicate and fitting of the averaged data revealed half-lives for the subunit exchange of 9.7 ± 2.2 min (average ± SEM) for the wildtype-MBP/wildtype-His combination and 7.4 ± 0.4 min for the wildtype-MBP/R882H-His combination. (**D**) Documentation of heterocomplex formation after pre-incubation of MBP-DNMT3AC (3AC-MBP) with His-DNMT3AC (3AC-His) or His-R882H (R882H-His). After the pre-incubation step, the proteins were purified over Ni-NTA beads. The specific co-purification of MBD-DNMT3AC indicated formation of mixed complexes with His-DNMT3AC and R882H-His.

To prepare for the kinetic analyses, we isolated the His-tagged wildtype and R882H proteins separately and confirmed that under our assay condition and with the used 30mer oligonucleotide substrate enzyme activity is linearly related to the enzyme concentration, both for wildtype DNMT3AC and for R882H (Fig. 3A). This finding is in agreement with our previous studies with wildtype DNMT3AC^28^. To investigate the dominant negative effect of the R882H mutation in mixed wildtype/R882H complexes, the purified wildtype and R882H proteins were mixed and incubated together for 30 min to allow for subunit exchange. In parallel, each protein preparation was separately pre-incubated under the same conditions. Afterwards, we measured the catalytic activities of all samples and compared the activity of the mixed sample with the activities of the individual subunits pre-incubated alone. However, we did not observe any difference between the activities of the mixed complexes when compared to the sum of the activities of the individual enzymes after equal treatment (Fig. 3B).

To confirm subunit exchange of the separately purified DNMT3AC wildtype and R882H subunits during the pre-incubation phase, wildtype DNMT3AC and R882H were cloned with His- and MBP-tags. All four proteins were purified by His- or MBP-affinity chromatography and the activity of the purified enzymes was determined. With MBP-tag, we observed approximately 65% residual activity of R882H (Fig. 2B,C), which is in agreement with the previous results obtained with His-tag purified enzyme. Moreover, we observed that MBP-tagged wildtype DNMT3AC and R882H both showed about 40% reduction in activity when compared with the corresponding His-tagged versions (Fig. 2D). Afterwards, Alpha-screen assays were conducted to investigate the subunit exchange of purified His- or MBP-tagged homomultimers (Fig. 3C). His-tagged wildtype DNMT3AC or R882H mutant were bound to Nickel chelate Alpha donor beads and MBP-tagged wildtype DNMT3AC to Anti-MBP acceptor beads. Both samples were mixed and incubated at ambient temperature and the Alpha signal determined for up to 45 min. Complex formation between the differently tagged subunits (presumably via the less stable RD interface) leads to a close approximation of the attached beads causing the emission of a detectable light signal. After mixing of His- and MBT-tagged wildtype DNMT3A, we indeed observed a clear increase in the Alpha-signal (Fig. 3C). After mixing of His-wildtype and MBP-R882H very similar kinetics of subunit exchange were observed. Fitting of the kinetics to an exponential curve revealed half-lives of 7–10 min in each case.

To confirm the conclusion of the Alpha-screen assays, that mixed complexes were obtained during the pre-incubation of separately purified enzyme preparations, we purified MBP-tagged wildtype DNMT3AC and pre-incubated it with purified His-tagged wildtype DNMT3AC or R882H. Afterwards, the protein complexes were purified over Ni-NTA beads (Fig. 3D). No MBP-tagged DNTM3AC was recovered in the absence of His-tagged proteins, showing the lack of unspecific interaction with the beads. In contrast, after pre-incubation with one of the His-tagged DNMT3AC proteins, similar amounts of MBP-tagged DNMT3AC were co-purified over the Ni-NTA beads indicating that mixed wildtype MBP-DNMT3AC were formed by wildtype His-DNMT3AC and His-R882H with comparable efficiencies.

All these data indicate that subunit exchange occurs during the pre-incubation phase of the enzyme activity assays described above. Based on the finding that pre-incubation of purified homomultimeric wildtype and R882H subunits did not lead to changes in activity although mixed wildtype/R882H complexes were formed, we conclude that a dominant negative effect of the R882H mutation could not be observed in this experiment.

### Purified mixed wildtype/R882H complexes do not show dominant negative effects of the R882H mutation

**Figure 4:**
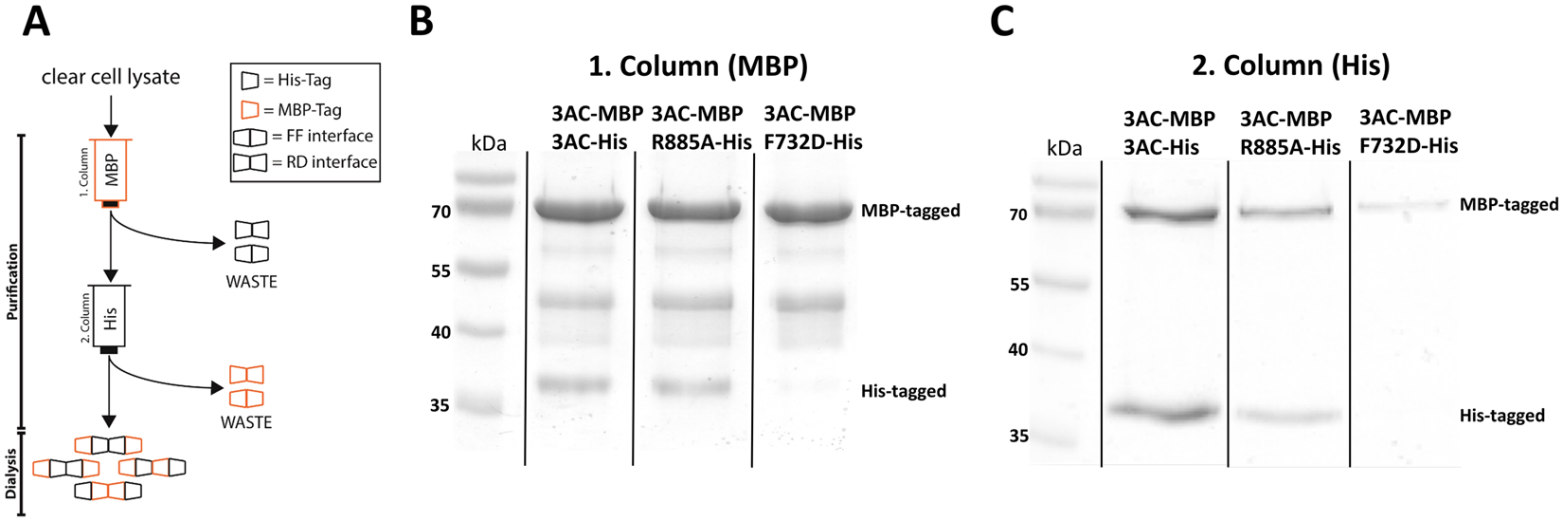
Characterization of the double-tag purification system of mixed His- and MBP-tagged DNMT3AC complexes. (**A**) Schematic representation of the purification of MBP- and His-tagged heteromultimeric DNMT3AC complexes by successive passage over MBP- and His- affinity columns. (**B**,**C**) Validation of the co-purification procedure. While mixed wildtype/wildype and wildtype/R885A complexes can be purified with good yield, the F732D mutation in the FF interface prevents the purification of complexes. In contrast, the R885A mutation in the RD interface did not change the His/MBP stoichiometry of the co-purified complexes. These results indicate that mainly His/MBP FF-interface heterodimers are purified using this protocol. For the original images of panels B and C, cf. Suppl. Fig. 1.

To further investigate the activity of wildtype/R882H complexes, we directly purified mixed complexes by applying a double tag purification strategy^29^. To this end, different DNMT3A subunits were co-expressed with His- and MBP-tags and two successive steps of His- and MBP-affinity chromatography were applied, allowing the purification of heterotypic complexes containing subunits with both tags. To establish this protocol, we have used DNMT3AC mutations, which were shown to disrupt the FF (F732D) and RD interface (R885 A)^15,17^. Note, that in the reference papers mouse DNMT3A numbering was used, such that the mutations were designated as F728D and R881A. These experiments revealed that the co-purification of mixed His-/MBP-tagged complexes with R885A mutation was possible, but purification of mixed complexes with the F732D mutation failed (Fig. 4). This result indicated that under the high salt conditions of this experiment mainly mixed heterodimers formed via the FF-interface were purified. This finding is in agreement with the observation that high salt buffers disrupt the RD interface contact^18^. During and after dialysis these mixed heterodimers form different kinds of heterotetramers and higher complexes via their RD interface.

**Figure 5:**
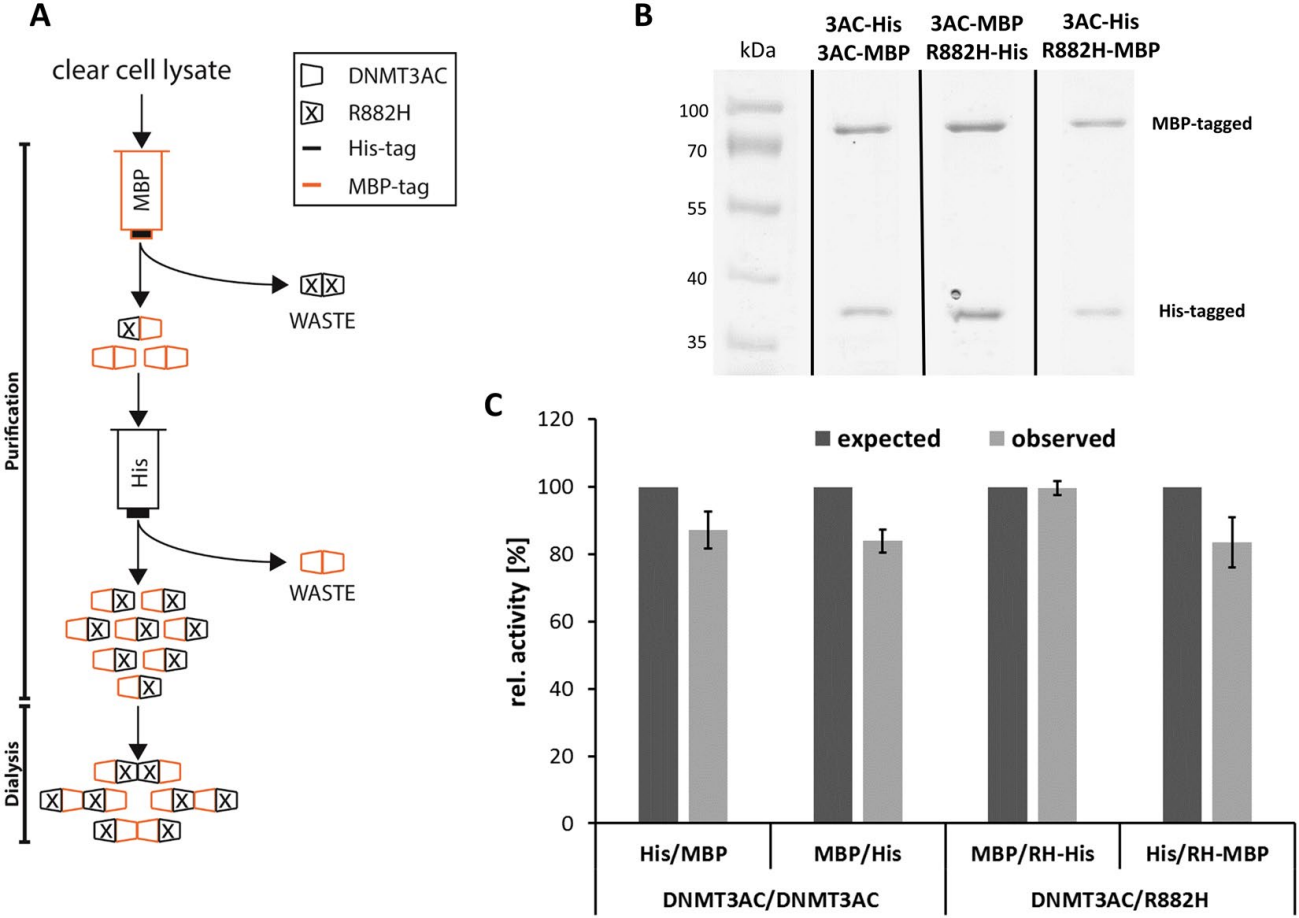
Purification and activity analysis of mixed R882H/wildtype DNMT3A complexes. (**A**) Schematic representation of the purification of mixed DNMT3AC/R882H complexes using a dual affinity approach. In this example, an MBP-wt/His-R882H complex is purified by successive passage over MBP- and His- affinity columns. (**B**) Example of purified His/MBP tagged mixed complexes. The image shows a Coomassie stained SDS-polyacrylamide gel. (**C**) Catalytic activities of purified His/MBP tagged heterotypic DNMT3AC complexes. On the left side, data for heterotypic His/MBP complexes containing two wildtype subunits are shown, on the right side, data for heterotypic wildtype/R882H complexes are displayed. The dark grey bars indicate expected activities corresponding to the sum of the activities of the two subunits, the light grey bars show the observed activities. Error bars indicate the SEM based on 3 independent experiments. For details of the analysis and the original image of panel B, cf. Suppl. Fig. 2.

Next, mixed wildtype and R882H complexes were purified using both combinations of tags and subunits (Fig. 5A). As a control, wildtype DNMT3AC with His- and MBP-tags was co-expressed and purified using the same protocol. The subunit composition of the purified protein preparations was analyzed by SDS-gel electrophoresis (Fig. 5B) and the exact amounts of both subunits were determined from stained gel images. Using the experimentally measured activities of the His- and MBP-tagged wildtype and R882H subunits determined above, expected theoretical activities of the mixed complexes were calculated based on the subunit concentration in the purified enzyme samples. These calculated activities incorporate all the effects of the R882H mutation and of both tags on the overall activity, but they assume that the R882H and wildtype subunits do not influence each other. A dominant negative effect of R882H in mixed complexes would cause a drop in the observed activity of mixed wildtype/R882H complexes below the expected activity level. We experimentally determined the enzymatic activity of the mixed complex enzyme preparations, but found no difference to the expected values (Fig. 5C). We conclude that biochemically purified heterotypic R882H/wildtype DNMT3A complexes did not reveal evidence for a dominant negative effect of the mutant subunit on the wildtype subunit in *in vitro* DNA methylation kinetics.

### Full length DNMT3A2 wildtype/R882H complexes do not show dominant negative effects of the R882H mutation

**Figure 6:**
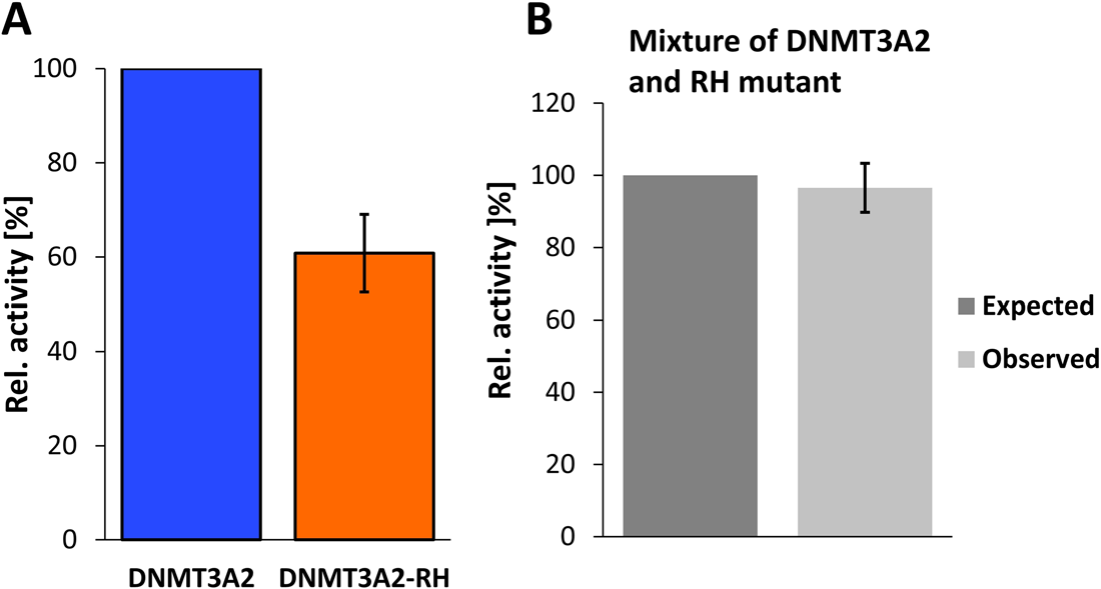
Effect of the RH mutation in the context of DNMT3A2. (**A**) Characterization of the activity of wildtype and mutant DNMT3A2 homomultimers. The error bar indicates the SEM based on 4 independent experiments. (**B**) Activity of mixed wildtype and mutant DNMT3A2 preparations after pre-incubation. The dark grey bar indicates the expected activity corresponding to the sum of the activities of the two subunit types, the light grey bar shows the observed activity. The error bar indicates the SEM based on 2 independent experiments.

To study the effect of the R882H mutation in the context of full-length DNMT3A, we have used the His-tagged murine DNMT3A2 protein and introduced the R878H mutation (corresponding to R882H in human DNMT3A). DNMT3A2 is a naturally occurring isoform of DNMT3A, which is expressed from an internal start site and lacks 219 N-terminal amino acids^30^. Wildtype and R878H DNMT3A2 proteins were purified after recombinant expression in *E. coli*. *In vitro* methylation experiments using identical concentrations of both enzymes showed that the R878H mutant has a residual activity of approximately 61% with the 30mer DNA substrate (Fig. 6A), which is very similar to the results obtained with the catalytic domain. To investigate potential dominant negative effects of the R878H mutation, we pre-incubated the wildtype and R878H DNMT3A2 proteins and determined the catalytic activity, which was then compared with the expected activity based on the contribution of the individual subunits pre-incubated under the same conditions. As shown in Fig. 6B, there was no detectable change in activity indicating the absence of dominant negative effects caused by the formation of mixed complexes *in vitro*.

## Discussion

Despite the fact that the R882H mutation in the RD interface of DNMT3A is highly prevalent in AML, its pathogenic mechanism has not been finally clarified at the molecular level^31^. Most previous publications reported a moderately reduced activity of the mutant, but a general loss-of-function mechanism is not in agreement with the strong enrichment of this particular mutation, the general absence of insertions and deletions in the mutational spectrum of *DNMT3A* and the heterozygous occurrence of the mutation accompanied by a wildtype *DNMT3A* allele. Russler-Germain *et al*. (2014) proposed a model in which the R882H mutation has a dominant negative effect in mixed wildtype/R882H complexes, which was based on biochemical evidence discussed below^22^. To investigate this dominant negative effect experimentally in greater mechanistic details, we formed mixed wildtype/R882H complexes by pre-incubation of separately isolated wildtype and R882H DNMT3AC subunits. However, no dominant negative effect was observed. This result is in agreement with data reported by Russler-Germain *et al*. (2014)^22^. To explain the apparent lack of a dominant negative effect in this experimental setting, they argued that subunit exchange may not be possible after mixing of separately purified enzyme preparations. However, in our work we experimentally confirmed that mixed complexes are formed. This observation is also in agreement with previous biochemical work by several groups, which demonstrated that mixing of separately purified DNMT3AC and DNMT3LC leads to subunit exchange and efficient formation of DNMT3AC/3LC complexes^17–19,26,32–35^. Similarly, we further show that mixing of mutant and wildtype full-length DNMT3A2 did not reveal dominant negative effects at the biochemical level.

We next have specifically purified mixed DNMT3A wildtype/R882H catalytic domain complexes and investigated their catalytic activities. To the best of our knowledge, our study for the first time reports the preparation of defined DNMT3A/R882H heterocomplexes which allows us to investigate potential dominant negative effects directly. However, similarly as in the experiments discussed in the last paragraph, no dominant negative effects were observed. This result is not in agreement with the reported results of Russler-Germain *et al*. (2014), but it is relevant to discuss some problems in the data interpretation of the previous publication. Russler-Germain *et al*. (2014) have determined the residual activity of R882H to be around 29% of wildtype DNMT3A, lower than results published by other labs (Table 1). Subsequently, they have co-expressed His-tagged wildtype DNMT3A and R882H and purified them together. They obtained a preparation containing about 50% of wildtype and R882H subunits, but observed an overall activity of only 12% and concluded that R882H has a dominant negative effect inhibiting the activity of wildtype subunits in mixed heterotetramers^22^. However, this biochemical result was surprising, because it is not compatible with a stochastic interface formation in which 25% wildtype/wildtype, 50% wildtype/R882H and 25% R882H/R882H complexes should be formed and a residual activity of at least 32.25% would have been expected (25% activity from the 25% wildtype homotypic complexes, which are fully active, plus 7.25% activity from the 25% R882H homotypic complexes) even if both subunits in mixed wildtype/R882H complexes were assumed to be completely inactive. Based on this it is likely, that for technical reasons the mixed enzyme preparation had a reduced activity, which was not related to the presence of wildtype and R882H subunits.

Our data presented in this work shed strong doubts on the biochemical dominant negative model based on the formation of mixed heterocomplexes, suggesting that additional evidence would be needed to substantiate it. Although one cannot rule out that enzymes expressed in human cells may behave differently, the biochemical dominant negative effect model is currently lacking supportive evidence, because of the technical concerns regarding the work of Russler-Germain *et al*. (2014) and the lack of biochemical dominant negative effects in purified mixed DNMT3A/R882H complexes reported here. Several groups have observed that R882H leads to changes in the cellular DNA methylation in cancer cells, often hinting towards a dominant negative effect in cells^22,24,36–38^. However, a dominant negative behavior of R882H (or other DNMT3A mutants^16^) in cells may be explained by several other mechanisms unrelated to a direct inhibition of wildtype subunits in heterocomplexes. In addition, cellular effects may be related to changes in the flanking sequence of R882H recently observed by us, which modulate the effects of R882H on DNMT3A activity^23^. Resolving these issues will be of high relevance to understand the pathogenic mechanism of the R882H mutation in AML.

## Methods

### Site-directed mutagenesis, protein expression and purification

Mutagenesis was performed using the megaprimer site-directed mutagenesis method^39^ and confirmed by restriction marker analysis and DNA sequencing. The C-terminal domain of human DNMT3A (Q9Y6K1.4) (amino acids 612–912) and its R882H mutant were cloned into pET28 + (Novagen) creating an N-terminal His_6_-tag fusion and into pMAL p2X (New England BioLabs) creating an N-terminal MBP-tag fusion. His-tagged DNMT3AC and DNMT3A2 and their mutants were overexpressed in BL21 (DE3) Codon + RIL *Escherichia coli* cells (Stratagene) and purified as described^28^. Briefly, cells were grown in TB medium until an A_600nm_ of 0.6 was reached and protein expression was induced for 12 h at 20 °C by addition of 0.5 mM isopropyl-1-thio-β-D-galactopyranoside. The proteins were purified at high micromolar concentrations using Ni–NTA agarose and stored in 20 mM HEPES pH 7.5, 200 mM KCl, 0.2 mM DTT, 1 mM EDTA and 10% glycerol at −80 °C. For purification of the MBP-tagged proteins, amylose resin matrix (New England Biolabs) was used and the proteins were eluted using 20 mM maltose and stored in 20 mM HEPES pH 7.5, 200 mM KCl, 0.2 mM DTT, 1 mM EDTA and 10% glycerol at −80 °C. Each protein was purified at least twice and the purity of the preparations was estimated to be >95% from Coomassie stained SDS gels. The concentrations of the proteins were determined by UV spectrophotometry and confirmed by densitometric analysis of Coomassie stained SDS–polyacrylamide gels.

### Purification of mixed DNMT3A/R882H complexes

For purification of the mixed complexes consisting of His-DNMT3AC/MBP-DNMT3AC, His-DNMT3AC/MBP-DNMT3AC-R882H and His-DNMT3AC-R882H/MBP-DNMT3AC, the corresponding subunits were co-overexpressed in BL21 (DE3) codon + RIL *Eschericha coli* cells (Stratagene). Cells were transformed with the pET28 + and pMAL vectors encoding for the corresponding DNMT3A wildtype or mutant and cultivated in the presence of Kanamycin and Ampicillin to ensure the presence of both plasmids. Cells were grown in TB medium containing both antibiotics until an A_600nm_ of 0.6 was reached and the protein expression was induced for 12 h at 20 °C by adding 0.5 mM isopropyl-1-thio-β-D-galactopyranoside. The mixed protein complexes were purified first over an amylose resin matrix (New England Biolabs) for binding MBP-tag containing protein complexes. The eluate was then used for a second purification step, using a nickel-NTA acid-agarose matrix for capturing all protein complexes which in addition to the MBP-tagged fusion proteins, contain His-tagged fusion proteins as well. The mixed complexes were stored in 20 mM HEPES/HCl pH 7.5, 200 mM KCl, 0.2 mM DTT, 1 mM EDTA, and 10% glycerol at −80 °C. The concentration and purity of the different mixed complex preparations was determined by UV absorption at 280 nm and by densitometric analysis of Coomassie BB stained SDS-polyacrylamide gels using ImageJ.

### Methyltransferase activity assay

The methyltransferase activity of DNMT3A was measured using a biotinylated double stranded 30-mer oligonucleotide (GAG AAG CTG GGA CTT CCG GGA GGA GAG TGC) containing a single CpG site as described^28^. Briefly, DNA methylation was measured by the incorporation of tritiated methyl groups from radioactively labeled AdoMet (Perkin Elmer) into the biotinylated substrate, using an avidin–biotin methylation plate assay^40^. The methylation reactions were carried out in methylation buffer (20 mM HEPES pH 7.5, 1 mM EDTA, 50 mM KCl, 0.05 mg/ml bovine serum albumin) at 37 °C using 2 µM DNMT3AC wildtype or mutant enzyme, 1 µM DNA and the reaction was started by adding 0.76 µM AdoMet. The initial slope of the enzymatic reaction was determined by linear regression.

### Subunit exchange experiments

To investigate the effect of the exchange of subunits on the catalytic activity of DNMT3AC, a mixture containing DNMT3AC-His and R882H-His (500 nM of each) was prepared and 1 µM of the standard 30mer substrate was added in methylation buffer not containing AdoMet. As control, 1 µM of the separate proteins were used and treated identically. After 30 min of pre-incubation at ambient temperature, the methylation reactions were started by addition of 0.76 µM radioactively labeled AdoMet. Afterwards, the methylation reactions were processed as described before.

Subunit exchanged and formation of heterotypic complexes was studied by Alpha-assay using His-tagged and MBP-tagged proteins. For His-tagged proteins, nickel chelate donor beads (PerkinElmer) were used and for MBP-tagged proteins anti-MBP AlphaLISA acceptor beads (PerkinElmer). Each protein (250 nM) was separately incubated for 20 min at RT with 20 µg/ml of the corresponding beads to allow for binding. At the start point of the experiment, the corresponding samples were combined and Alpha-signal measurements were taken after 0, 5, 10, 15, 30 and 45 min using an EnSpire Alpha Plate Reader (PerkinElmer). Signals obtained after incubation of the corresponding His-tagged DNMT3 proteins with untagged MBP or empty beads were used as control. Since it is known that the concentration of monomeric DNMT3 species is low^17,19^ the rate limiting step for subunit exchange is the dissociation of existing complexes. Therefore, the data were fitted by non-linear regression to a monomolecular decay function using the solver function of Microsoft Excel to determine the half-lives of subunit exchange.

Subunit exchanged and formation of heterotypic complexes was also studied by co-purification with Ni-NTA acid-agarose beads. For this, MBP-DNMT3AC was pre-incubated with His-DNMT3AC or His-R882H in incubation buffer (25 mM Tris/HCl pH 7.5, 100 mM KCl, 5 mM MgCl_2_, 10% glycerol, 0.1% NP-40, 200 µM PMSF) and the pre-incubated mixtures were bound to 100 µl Ni-NTA beads, washed 3 times with wash buffer (25 mM Tris/HCl pH 7.5, 300 mM KCl, 5 mM MgCl_2_, 10% glycerol, 0.1% NP-40, 200 µM PMSF) and afterwards eluted in 300 µl of wash buffer supplemented with 220 mM Imidazole. The eluted proteins were TCA precipitated and loaded on SDS polyacrylabmide gels stained with Coomassie BB. Identical reactions without His-tagged protein were carried out as controls.

## Electronic supplementary material

Supplemental information

## Data Availability

All data generated or analyzed during this study are included in this published article (and its Supplementary Information files).
