# Supplementary material for: The DNMT3A R882H mutation does not cause dominant negative effects in purified mixed DNMT3A/R882H complexes
Source: Sci Rep. 2018 Sep 5;8:13242. doi: 10.1038/s41598-018-31635-8 (PMC6125428; doi:10.1038/s41598-018-31635-8)
Supplement: Supplementary file 1 — Supplemental information [file 41598_2018_31635_MOESM1_ESM.pdf]

The DNMT3A R882H mutation does not cause dominant negative effects in purified mixed DNMT3A/R882H complexes

Max Emperle, Michael Dukatz, Stefan Kunert, Katharina Holzer, Arumugam Rajavelu, Renata Z. Jurkowska, & Albert Jeltsch*

Supplemental information

Supplemental Figure 1. Original images of Fig. 4B and C.

Supplemental Figure 2. Details of the analysis of the data shown in Fig. 5C. Gel 2 represents the original image of Fig. 5B.

Suppl. Fig. 1: Original images of Fig. 4B and C.

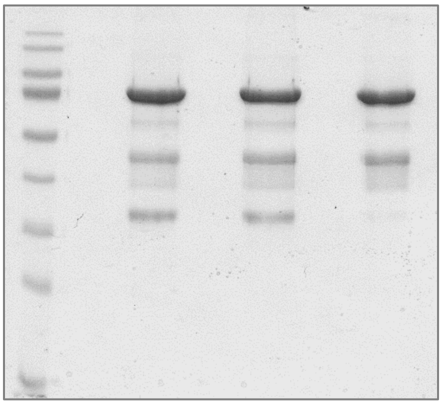

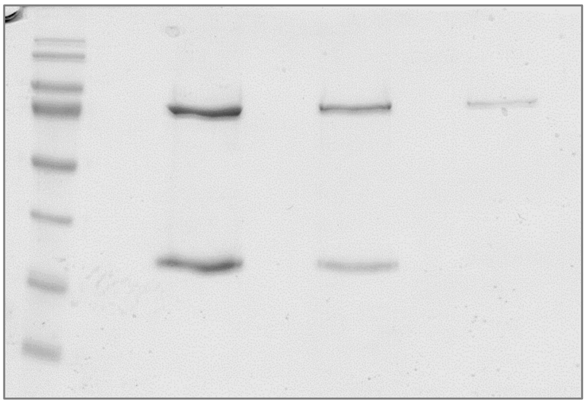

Supplemental Figure 2. Details of the analysis of the data shown in Fig. 5C. Gel 2 represents the original image of Fig. 5B.

Gel 1

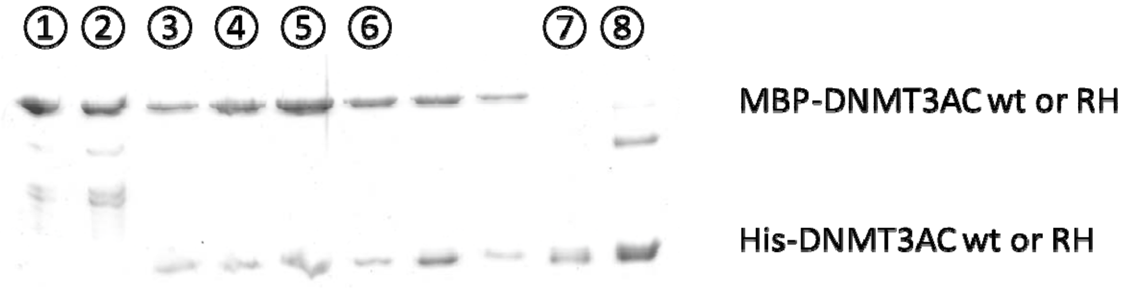

Lane	Lane 1	Lane 2	Lane 3	Lane 4	Lane 5	Lane 6	Lane 7	Lane 8	
Protein 1	Wt-MBP	RH-MBP	Wt-His	Wt-MBP	Wt-His	Wt-His	Wt-His	RH-His	
Protein 2			Wt-MBP	Wt-His	RH-MBP	RH-MBP			
Relative pixel intensities of the protein bands		(Intensities of the MBP proteins were divided by 2.1 to consider the larger mol. weight.)							
Int. His			0.51	0.44		0.28	1.00	2.5	
Int. MBP	1.25	1.09	0.56	0.76		0.37			
Activity	22.88	11.86	27.69	31.53	Lane 5 could not be analysed	10.50	45.35	46.73	CPM/min
Rel. activities of reference proteins	18.31	10.88					45.35	18.69	Activity/Protein amount
Expected activities of His-proteins			23.13	19.95		12.70			Protein amount * rel. activity of His-reference protein
Expected activities of MBP-proteins			10.25	13.99		4.02			Protein amount * rel. activity of MBP-reference protein
Sum of expected activity			33.38	33.95		16.72			
Observed/expected activity			0.83	0.93		0.63			

Gel 2

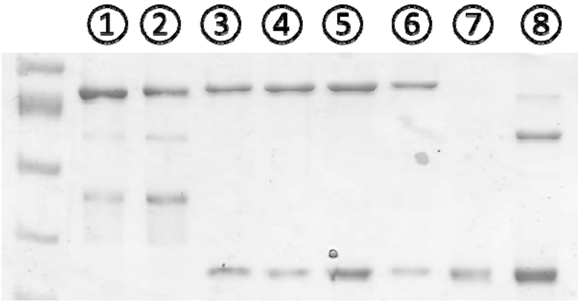

Lane	Lane 1	Lane 2	Lane 3	Lane 4	Lane 5	Lane 6	Lane 7	Lane 8	
Protein 1	Wt-MBP	RH-MBP	Wt-His	Wt-MBP	Wt-MBP	Wt-His	Wt-His	RH-His	
Protein 2			Wt-MBP	Wt-His	RH-His	RH-MBP			
Relative pixel intensities of the protein bands		(Intensities of the MBP proteins were divided by 2.1 to consider the larger mol. weight.)							
Int. His			1.08	0.55	1.87	0.28	1.00	2.85	
Int. MBP	1.56	1.07	0.94	1.05	1.21	0.65			
Activity	26.36	11.33	60.51	44.58	87.49	26.02	61.70	114.38	CPM/min
Rel. activities of reference proteins	16.90	10.59					61.70	40.13	Activity/Protein amount
Expected activities of His-proteins			66.64	33.94	75.05	17.28			Protein amount * rel. activity of His-reference protein
Expected activities of MBP-proteins			15.89	17.74	12.81	6.88			Protein amount * rel. activity of MBP-reference protein
Sum of expected activity			82.53	51.68	87.86	24.16			
Observed/expected activity			0.73	0.86	1.00	1.08			

Gel 3

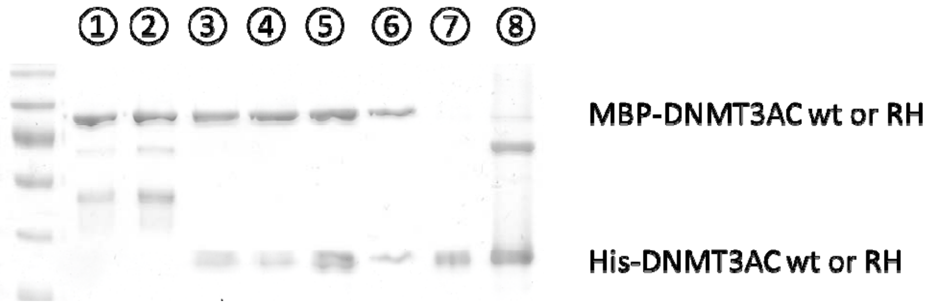

Lane	Lane 1	Lane 2	Lane 3	Lane 4	Lane 5	Lane 6	Lane 7	Lane 8	
Protein 1	Wt-MBP	RH-MBP	Wt-His	Wt-MBP	Wt-MBP	Wt-His	Wt-His	RH-His	
Protein 2			Wt-MBP	Wt-His	RH-His	RH-MBP			
Relative pixel intensities of the protein bands		(Intensities of the MBP proteins were divided by 2.1 to consider the larger mol. weight.)							
Int. His			0.65	0.49	1.41	0.41	1.00	1.94	
Int. MBP	0.67	0.64	0.61	0.72	0.80	0.41			
Activity	21.40	15.55	74.37	44.67	135.89	33.78	78.54	152.23	CPM/min
Rel. activities of reference proteins	31.94	24.29					78.54	78.47	Activity/Protein amount
Expected activities of His-proteins			51.05	38.48	110.64	32.20			Protein amount * rel. activity of His-reference protein
Expected activities of MBP-proteins			19.49	23.00	25.55	9.96			Protein amount * rel. activity of MBP-reference protein
Sum of expected activity			70.53	61.48	136.20	42.16			
Observed/expected activity			1.05	0.73	1.00	0.80			
